# Effects of Graphene-Based Nanomaterials on Anaerobic Digestion of Thermally Hydrolyzed Municipal Sewage Sludge

**DOI:** 10.3390/ma18153561

**Published:** 2025-07-29

**Authors:** Luiza Usevičiūtė, Tomas Januševičius, Vaidotas Danila, Mantas Pranskevičius

**Affiliations:** Research Institute of Environmental Protection, Vilnius Gediminas Technical University, LT-10223 Vilnius, Lithuania; tomas.janusevicius@vilniustech.lt (T.J.); vaidotas.danila@vilniustech.lt (V.D.); mantas.pranskevicius@vilniustech.lt (M.P.)

**Keywords:** wet-type anaerobic digestion, thermally hydrolyzed sewage sludge, graphene nanoplatelets, graphene oxide nanosheets, biogas, methane, kinetic modeling, maximum methane potential

## Abstract

In this study, the effects of graphene-based nanomaterials—specifically graphene nanoplatelets (GNPs) and graphene oxide (GO) nanosheets—on methane (CH_4_) production during anaerobic digestion (AD) of thermally hydrolyzed sewage sludge were investigated. Anaerobic digestion was carried out over a 40-day period under mesophilic conditions in batch digesters with a volume of 2.65 L. The influence of various dosages of GNPs and GO nanosheets on methane yields was assessed, including a comparison between GNPs with different specific surface areas (320 m^2^/g and 530 m^2^/g). The highest CH_4_ yield (194 mL/g-VS_added_) was observed with a GNP dosage of 5 mg/g-TS and a surface area of 530 m^2^/g, showing an increase of 3.08% compared to the control. This treatment group had the greatest positive effect also on the degradation of organic matter, with total solids (TS) and volatile solids (VS) removal reaching 34.35% and 44.18%, respectively. However, the GO dosages that significantly decreased cumulative CH_4_ production were determined to be 10–15 mg/g-TS. Graphene oxide at dosages of 10 and 15 mg/g-TS reduced specific cumulative CH_4_ yields by 4.03% and 5.85%, respectively, compared to the control, indicating CH_4_ yield inhibition. This lab-scale study highlights the potential for integrating GNPs into full-scale, continuously operated wastewater treatment anaerobic digesters for long-term use in future applications.

## 1. Introduction

Anaerobic digestion (AD) is a well-established technology for treating sewage sludge (SS), converting organic material into methane-rich biogas, which can be used for energy recover in wastewater treatment plants (WWTPs) [1]. Anaerobic digestion consists of four stages, with the final stage being methanogenesis, during which methane (CH_4_) is produced. Traditional AD encounters specific challenges, such as the build-up of volatile fatty acids (VFAs), which can be harmful to methanogenic microorganisms [2]. The AD process can be accelerated by enhancing direct interspecies electron transfer (DIET), which improves both the rate and efficiency of AD, resulting in increased CH_4_ production, higher organic conversion rates, reduced VFA concentrations, and a shorter start-up period. According to a previous study, the lag time for the start-up of thermophilic AD was reduced by 40% in reactors supplemented with conductive materials [3].

Recent studies have revealed that certain predominant methanogens (specifically *Methanosaeta* and *Methanosarcina* species) in methanogenic digesters are capable of receiving electrons from electron-producing microorganisms (for example, *Geobacter*) either directly through DIET or indirectly via a combination of biological and abiological electron transfer components, such as conductive materials [4,5]. The cells form biological electrical connections using their conductive pili, which function as nanowires. More recently, it has been confirmed that conductive materials such as carbon cloth, biochar, granular activated carbon (GAC), graphite, and magnetite can perform a similar role to pili by facilitating the electrical connections necessary for syntrophic metabolism [6]. One study showed that GAC was up to 1500 times more conductive than *Geobacter* co-culture aggregate (3000 µS/cm and 2 µS/cm, respectively). As a result, it enhanced electron transfer to *Methanosaeta* or *Methanosarcina* and stimulated the conversion of organic wastes to CH_4_ [7]. It was suggested that GAC promoted methanogenesis by providing electrical connection between cells (bacteria involved in degradation of organic compounds and CH_4_-producing bacteria).

Carbonaceous nanoparticles (CNPs) are used as additives in AD due to their outstanding mechanical and thermal durability and high electrical and thermal conductivity [8]. Among the different CNPs, graphene nanoplatelets (GNPs) have attracted increased interest because of their interesting mechanical and electrical properties, such as high strength, specific surface area (SSA), and electron mobility. Such properties enhance bacterial extracellular electron transfer and DIET, and ultimately help to improve CH_4_ production efficiency in AD systems [9,10]. Graphene is a highly conductive nanomaterial composed of a two-dimensional sheet of carbon atoms arranged in a hexagonal honeycomb lattice, while graphene oxide (GO) consists of graphene-based lattices containing a variety of oxygenated functional groups, such as epoxy, ketone carbonyl, carboxyl, and hydroxyl [9,11,12]. Contrary to graphene, there have been reports that GO demonstrates a negative effect on methanogenic activity due to membrane cell damage, oxidative stress, or wrapping isolation [1].

Numerous studies have shown that CNP additives are suitable for increasing CH_4_ production due to their enhancement of DIET. For example, Tian et al. [13] demonstrated that nanographene dosages of 30 mg/L and 120 mg/L significantly increased CH_4_ production rate by 17% and 51.4%, respectively, using synthetic wastewater and glucose as substrate and activated sludge as the inoculum at 35 ± 1 °C. Additionally, an increase in *Geobacter* abundance was observed in the presence of graphene, indicating that DIET could be enhanced. It is well documented that *Geobacter* species are among the most commonly encountered bacteria that act as electron donors in DIET for methanogens [14]. Research conducted by Lin et al. [15] also showed that graphene particles (size of 5~10 µm and thickness of nanoplatelets of 4–20 nm) enhanced CH_4_ yield by 25% and production rate by 19.5% using ethanol as a substrate and activated sludge as the inoculum. It is also worth mentioning that this study determined that graphene addition resulted in significantly higher CH_4_ production compared to activated carbon and this was ascribed to the higher electrical conductivity of graphene and the higher electron transfer efficiency in DIET. Another study performed by Wang et al. [16] compared the influence of graphite, graphene, and GO on the performance of anaerobic co-digestion of SS and food waste and found that graphene enhanced CH_4_ production and organic degradation the most (by 36.1% and 23.1%, respectively). All of these studies showed that the addition of conductive graphene enhanced methanogenesis by promoting DIET between syntrophic bacteria and methanogens. However, when considering the addition of GO in AD systems, contradictory results can be found in the literature. Some studies highlighted that GO addition has the potential to improve CH_4_ yields. For example, Kundu et al. [17] achieved a 1.35-fold-higher CH_4_ yield in a GO-amended AD system with fermented Assam lemon waste compared to the control. Wang et al. [16] reported that an AD system amended with GO produced a 17.6% higher cumulative CH_4_ yield compared to the control. However, there are some studies that showed an inhibitory effect of GO on methanogenesis. Anaerobic digestion of waste-activated sludge with GO demonstrated a decrease in cumulative CH_4_ production by 7.6% and 12.6% at dosages of 0.054 mg/mg-VS and 0.108 mg/mg-VS, respectively, compared to the control [18]. A similar inhibitory effect of GO on CH_4_ production was observed during AD of swine manure, when CH_4_ production was reduced by 13.1% and 17.1% at GO dosages of 5 mg/L and 500 mg/L, respectively [19]. In summary, based on the studies conducted so far, it can be stated that the main challenge remains identifying the appropriate dosage and SSA of graphene-based nanomaterials that would have a positive effect on CH_4_ yield.

There has been limited research into whether graphene-based nanomaterials can enhance the AD of SS treated by thermal hydrolysis. To date, most studies have focused on evaluating the effects of graphene materials on the AD of small-molecule substrates (such as glucose and ethanol) and more complex substrates (such as swine manure and mixtures of food waste and SS). This is one of the first studies to investigate the effect of graphene-based nanomaterials on biogas and CH_4_ production during the AD of thermally hydrolyzed sewage sludge (THSS). The combination of pretreated sludge and graphene-based additives presents a novel approach to enhancing AD performance.

It was hypothesized that the addition of GNPs to THSS would shorten the lag phase and enhance cumulative CH_4_ yield during AD by facilitating DIET. In contrast, the addition of high dosages of GO was expected to have an inhibitory effect by increasing the lag phase, while also reducing the CH_4_ production rate and cumulative CH_4_ yield.

Therefore, this study aimed to evaluate the effects of two types of graphene-based nanomaterials, specifically GNPs with different surface areas (320 m^2^/g and 530 m^2^/g) and GO, on the AD of THSS. The research was performed in 2.65 L batch-mode glass reactors with a working volume of 2 L using a mixture of THSS as a substrate and anaerobically digested SS as the inoculum with supplementation of respective graphene-based nanomaterials. The objectives of this study are to (1) compare biogas and CH_4_ production over time in the AD of THSS with different dosages of GNPs and GO; (2) determine total solids (TS), volatile solids (VS), and chemical oxygen demand (COD) removal efficiencies; and (3) apply the modified Gompertz model (MGM) to simulate and evaluate the methane production kinetics during AD of THSS treated with graphene-based nanomaterials.

## 2. Materials and Methods

### 2.1. Graphene-Based Nanomaterials, Substrate, and Inoculum

Commercially available GNPs and GO were purchased from Nanografi, Ankara, Turkey. GNPs with SSAs of 320 m^2^/g and 530 m^2^/g, as well as GO with an SSA of 420 m^2^/g, were selected to assess their impact on methanogenesis. Graphene nanoplatelets of both surface areas have the following characteristics: purity 99.9%, a thickness of 3 nm, and a diameter of 1.5 µm. Meanwhile, GO consists of 2–5 stacked layers and has an average diameter of 4.5 µm. Stock suspensions of GNPs and GO were prepared by dispersing 20 g of material in 1 L of deionized water, followed by sonication (35 kHz) for 30 min at room temperature.

Thermally hydrolyzed sewage sludge was collected as the substrate from a local WWTP located in Vilnius, Lithuania. The thermal hydrolysis pretreatment was carried out at 150 °C, 5–5.5 bar pressure, and a treatment time of 30 min. Inoculum sludge was collected from an anaerobic digester located in the same WWTP. Before being characterized and used in AD, THSS was allowed to cool to room temperature [20]. The characteristics of the sludge samples are shown in Table 1. It can be seen that the TS content in the sludge mixture was 7.38%, with the VS content at 3.62%. Previous study has shown that graphene-based nanomaterials may enhance the degradation of organic matter in wet AD systems where TS is below 10% [21].

### 2.2. Experimental Setup

**Figure 1:**
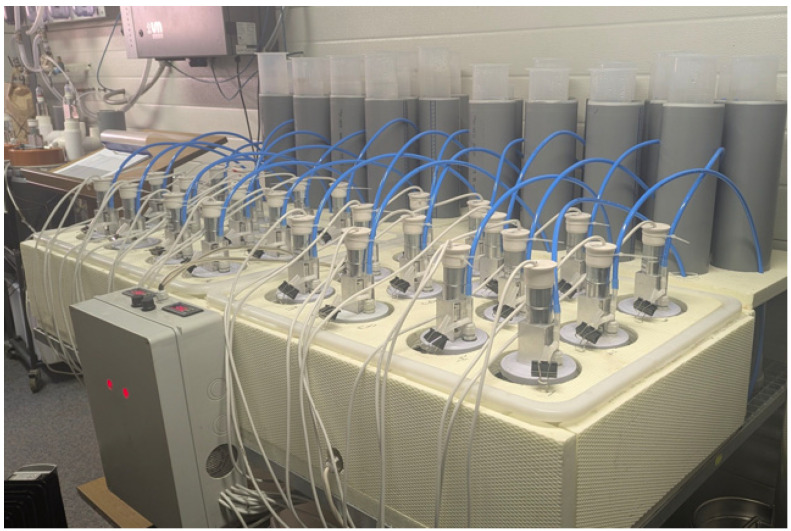
Laboratory setup for anaerobic digestion trials.

The AD tests were carried out in batch mode using an experimental setup consisting of 20 glass reactors (Figure 1). Each reactor had a total volume of 2.65 L and a working volume of 2 L. A substrate-to-inoculum ratio of 2:1 (volume basis) and 3:1 (VS basis) was applied. The reactors were divided into four main treatment groups: a control group without any additives (reactor B0); a group supplemented with GNPs with a surface area of 320 m^2^/g (reactors B1–B3); a group with GNPs with a surface area of 530 m^2^/g (reactors B4–B6); and a group with GO (reactors B7–B9) (Table 2). The dosages of GNPs and GO applied in this experiment were 5.0 mg/g-TS, 10 mg/g-TS, and 15 mg/g-TS. Each treatment condition was tested in duplicate. The reactors were maintained at 37.0  ±  1.0 °C using a temperature-controlled water bath throughout the 40-day operation period. During the AD process, the reactors were continuously stirred at a speed of 120 rpm [22]. Biogas volume and CH_4_ concentration were recorded daily.

### 2.3. Analytical Methods

The TS and VS contents were determined using standard procedures outlined by APHA [23]. Samples were oven-dried at 103–105 °C to a constant weight to determine TS content [23,24]. To determine VS content, the dried samples were combusted in a muffle furnace at 550 °C for 3 h. The COD was determined using a Lovibond MD100 colorimeter (Tintometer GmbH, Dortmund, Germany). A digital pH meter (SevenMulti™, Mettler Toledo GmbH, Schwerzenbach, Switzerland) was used to measure pH. Electrical conductivity was measured using a conductometer (inoLab 740, WTW, Weilheim, Germany). The concentrations of elements (C and N) in the samples were analyzed using an elemental analyzer (EuroEA3000, EuroVector, Pavia, Italy). The functional groups of the digested samples were evaluated using a Fourier transform infrared (FTIR) spectrometer (Invenio R, Bruker, Billerica, MA, USA). The spectral data were collected in the range of 400 to 4000 cm^−1^. The morphology of GNPs and GO nanosheets was characterized by using scanning electron microscopy (SEM) (FlexSEM 1000 II, Hitachi High-Tech Corporation, Hitachinaka, Japan). To ensure high-resolution imaging, graphene-based nanomaterial powder samples were dispersed in ethanol. Then dried nanomaterials were analyzed using an acceleration voltage of 15 kV, a working distance of 5 mm, and a magnification level of 5000× [25]. Energy-dispersive spectroscopy (EDS) analysis was performed using an EDS system (Oxford Instruments NanoAnalysis, High Wycombe, UK) integrated with an SEM to examine the chemical composition of the nanomaterials [26]. The concentration of CH_4_ was measured using a portable gas analyzer (GFM 406, Gas Data Ltd., Coventry, UK). Daily measurements of biogas volume were carried out with 2 L graduated cylinders.

### 2.4. Kinetic Analysis

Cumulative CH_4_ production data were fitted to the MGM to assess AD performance and estimate kinetic parameters for each treatment group [3]. The MGM equation is expressed as follows:

(1) $$M(t)=P_{max}\times exp\left\{-exp\left[\frac{R_{max}\times e}{P_{max}}\times \left(\lambda -t\right)+1\right]\right\},$$

where *M*(*t*)—specific cumulative CH_4_ production at time *t*, in mL/g-VS_added_; *P_max_*—maximum CH_4_ potential, in mL/g-VS_added_; *R_max_*—maximum CH_4_ production rate, in mL/g-VS_added_/d; *e*—exponential constant (=2.7182); *λ*—lag phase period, in days; and *t*—time, in days. The MGM is a sigmoidal function originally developed to describe bacterial growth, characterized by three distinct phases: a lag phase, an exponential growth phase, and a stationary phase [27]. This model is based on the assumption that growth is slower at both the initial and final stages, forming a sigmoid-shaped curve [28]. The MGM, commonly employed in the study of CH_4_ accumulation, is considered the most reliable model for predicting CH_4_ production [29].

The kinetic model parameters, such as *P_max_*, *R_max_*, and *λ*, were estimated using a cost function aimed at minimizing the sum of squared errors between the model-predicted values and the experimentally observed data [30]. For this optimization, the Generalized Reduced Gradient nonlinear solver in Microsoft Excel was employed. Subsequently, the experimental and predicted data points for *P_max_* were plotted against each other to assess the predictive performance of the MGM. The effectiveness of the diagram was evaluated based on how closely the data points clustered around the equality line and their deviation from the 45° reference line [31].

### 2.5. Calculations and Statistical Analysis

All AD experiments were performed in duplicate. The physicochemical parameters of the sludge samples (Table 1) were determined in triplicate. The results are presented as mean values ± standard deviation. Additionally, one-way analysis of variance (ANOVA) was used to evaluate statistically significant differences (*p* < 0.05) in biogas and CH_4_ production between the control and the groups treated with graphene-based nanomaterials [32]. Statistical analysis was performed using Microsoft Excel 2108.

Biogas and CH_4_ production for all treatment groups were expressed relative to the VS added to the reactors. Specific cumulative biogas yield (SCBY) and specific cumulative methane yield (SCMY) were calculated using the following equations [33]:

(2) $$SCBY=\frac{CVB}{gVS},$$

(3) $$SCMY=\frac{CVM}{gVS},$$

where SCBY—specific cumulative biogas yield, in mL/g-VS_added_; *CVB*—cumulative volume of biogas, in mL; *gVS*—amount of volatile solids introduced into each reactor, in g; SCMY—specific cumulative CH_4_ yield, in mL/g-VS_added_; and *CVM*—cumulative volume of CH_4_, in mL. Additionally, TS, VS, and COD—parameters indicative of organic matter decomposition—were also evaluated. The following Equations (4) and (5) were used to calculate TS and VS [32]:

(4) $$TS=\frac{S2-C}{S1-C}\times 100\%,$$

(5) $$VS=\frac{\left(S2-C)-(S3-C\right)}{S1-C}\times 100\%,$$

where *C*—weight of dish, in g; *S*2—weight of dried residue + dish, in g; *S*1—weight of wet sample + dish, in g; and *S*3—weight of ash after ignition + dish, in g.

Equation (6) was used to calculate the percentage reductions in TS, VS, and COD for each treatment group [21,34]:

(6) $$Removal\;Efficiency(TS,VS,COD)=\frac{{Value}_{0}\;-\;{Value}_{f}}{{Value}_{0}}\times 100\%,$$

where *Value*_0_—initial concentration of TS, VS, or COD fed into the reactors at the start of AD, in g/L; and *Value_f_*—final concentration of TS, VS, or COD remaining after AD, in g/L.

After generating the predicted data using the MGM, they were plotted against the experimental data, and the coefficient of determination (*R*^2^), root mean square error (*RMSE*), and normalized root mean square error (*NRMSE*) values were subsequently calculated. Equations (7)–(9) were used to calculate *R*^2^, *RMSE*, and *NRMSE*, respectively [35,36]:

(7) $$R^{2}=1-\frac{\sum {\left(Y_{i.exp}-Y_{i,cal}\right)}^{2}}{\sum {\left(Y_{i,exp}-\bar{Y}\right)}^{2}},$$

(8) $$RMSE=\sqrt{\frac{1}{n}\sum_{i=1}^{n}{\left(Y_{i,exp}-Y_{i,cal}\right)}_{i}^{2}},$$

(9) $$NRMSE=\left[\frac{RMSE}{Y_{max}-Y_{min}}\right]\times 100,$$

where *RMSE*—the root mean square error, in mL/g-VS_added_; *Y_i,exp_*—observations (experimental values used for simulations), in mL/g-VS_added_; *Y_i,cal_*—predictions (calculated values), in mL/g-VS_added_; *n*—number of observations; $\bar{Y}$—mean of observations, in mL/g-VS_added_; *NRMSE*—normalized *RMSE*, in %; *Y_max_*—maximum observed value of methane production, in mL/g-VS_added_; and *Y_min_*—minimum observed value of methane production, in mL/g-VS_added_.

## 3. Results and Discussion

### 3.1. Characterization of Graphene and Graphene Oxide

**Figure 2:**
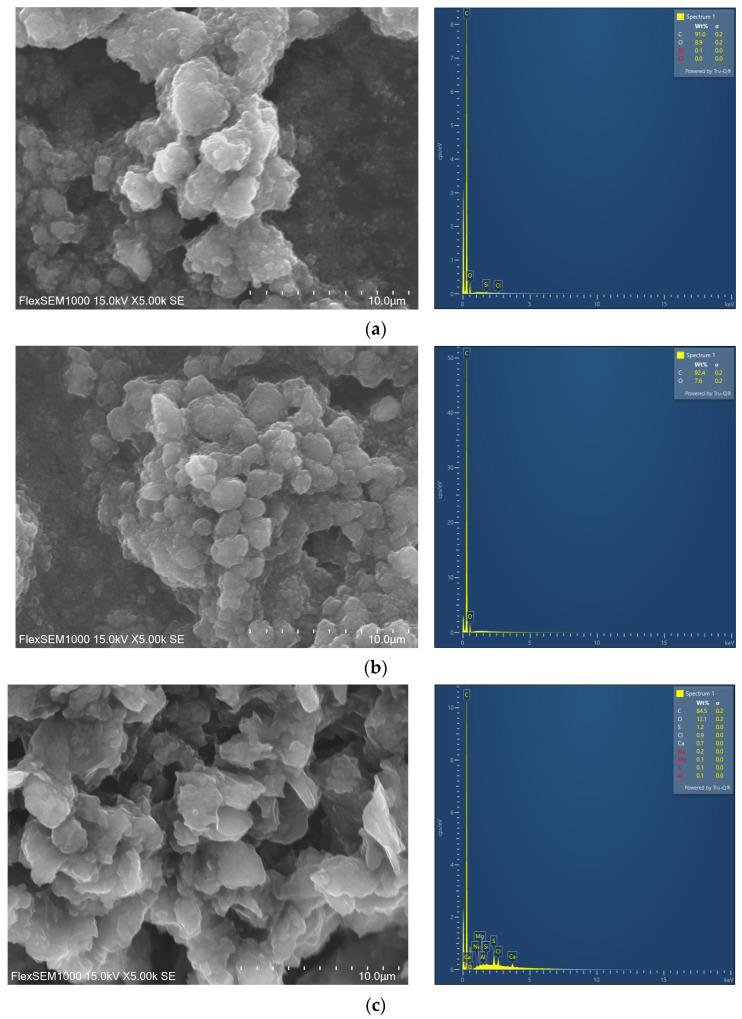
Scanning electron microscopy (SEM) images and corresponding energy-dispersive spectroscopy (EDS) data of (**a**) graphene nanoplatelets, 320 m^2^/g; (**b**) graphene nanoplatelets, 530 m^2^/g; and (**c**) graphene oxide nanosheets, 420 m^2^/g.

Figure 2 shows SEM images (5000× magnification) and EDS data of commercial GNPs with SSAs of 320 m^2^/g (Figure 2a) and 530 m^2^/g (Figure 2b), as well as commercial GO samples (Figure 2c). It can be seen that both types of GNPs exhibit a similar morphology. The GNPs of both SSAs are composed of thin graphene sheets stacked in multiple layers and arranged in a planar manner. The lateral size of GNPs with an SSA of 320 m^2^/g ranges from 1.0 µm to 3.2 µm, with a mean diameter of about 2.0 µm, whereas for GNPs with an SSA of 530 m^2^/g, the lateral size ranges from 1.3 µm to 2.6 µm, with a mean diameter of 1.8 µm. When compared with GNPs, it can be observed that GO sheets are less densely stacked and exhibit more random aggregation. In addition, GO sheets appear more wavy, which could be attributed to the presence of oxygen-containing functional groups [37]. The lateral size of GO ranges from 2.2 µm to 6.0 µm, with a mean diameter of about 4.0 µm.

EDS analysis was performed to determine the surface elemental composition of the GNPs and GO used in the study. All graphene-based nanomaterials primarily consisted of carbon (C) and oxygen (O) (Figure 2). For GNPs with an SSA of 320 m^2^/g, the C and O contents were 91.0% and 8.9%, respectively, whereas for GNPs with an SSA of 530 m^2^/g, the C and O contents were 92.4% and 7.6%, respectively. Graphene oxide exhibited C content of 84.5% and O of 12.1%. In addition, GO contained trace impurities such as S, Cl, Ca, Na, Mg, Si, and Al, which likely originated from the chemical reagents used during the oxidation process. A study by Méndez-Lozano et al. [38] similarly reported the presence of trace impurities, including Si, S, Cl, and K, in GO synthesized using an environmentally friendly modified Hummers method.

### 3.2. Effects of Graphene and Graphene Oxide on AD Performance

**Figure 3:**
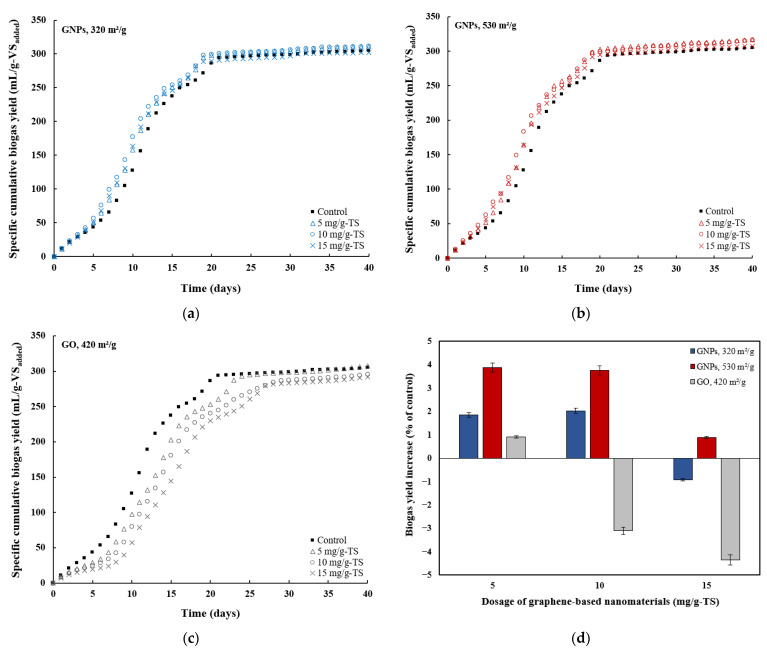
Specific cumulative biogas yield during anaerobic digestion of thermally hydrolyzed sewage sludge amended with different dosages of (**a**) graphene nanoplatelets, 320 m^2^/g; (**b**) graphene nanoplatelets, 530 m^2^/g; (**c**) graphene oxide nanosheets, 420 m^2^/g; and (**d**) increase in biogas yield for the different dosages of graphene-based nanomaterials.

Anaerobic digestion batch tests were carried out over 40 days in reactors supplemented with graphene-based nanomaterials, alongside a control reactor, to monitor biogas and CH_4_ production. At the end of the AD process, the SCBY reached 304.9 mL/g-VS_added_ in the control reactor, and 310.6 mL/g-VS_added_, 311.1 mL/g-VS_added_, and 302.1 mL/g-VS_added_ in the reactors to which GNPs with SSA of 320 m^2^/g were added at dosages of 5 mg/g-TS, 10 mg/g-TS, and 15 mg/g-TS, respectively (Figure 3a). It can be seen that the highest dosage of GNPs with the smaller SSA had a negligible effect on the total biogas yield. Meanwhile, in the reactors where GNPs with a higher SSA (530 m^2^/g) were added at dosages of 5 mg/g-TS, 10 mg/g-TS, and 15 mg/g-TS, the SCBY reached 316.7 mL/g-VS_added_, 316.4 mL/g-VS_added_, and 307.6 mL/g-VS_added_, respectively (Figure 3b). The addition of GNPs with higher SSA at 5 mg/g-TS and 10 mg/g-TS resulted in 3.87% and 3.76% increases in SCBY compared to the control (Figure 3d). When comparing the effects of GNPs with different SSAs on SCBY, the GNPs with a higher surface area (530 m^2^/g) at dosages of 5 mg/g-TS and 10 mg/g-TS were slightly more effective at enhancing biogas production compared to those with a surface area of 320 m^2^/g. Meanwhile, in the reactors where GO was added at dosages of 5 mg/g-TS, 10 mg/g-TS, and 15 mg/g-TS, the SCBYs reached 307.7 mL/g-VS_added_, 295.5 mL/g-VS_added_, and 291.7 mL/g-VS_added_, respectively (Figure 3c). Elevated GO dosages (10 mg/g-TS and 15 mg/g-TS) had a significant inhibitory effect on biogas production, with SCBYs being 3.11% and 4.35% lower, respectively, compared to the control. In contrast, the lowest GO dosage of 5 mg/g-TS had a negligible effect on cumulative biogas yield, resulting in a 0.92% increase in SCBY compared to the control. Several studies have also reported a negative impact of graphene and GO on biogas production [18,19,39,40]. Lin et al. [39] found that a 2 g/L dosage of graphene decreased the CH_4_ yield from 189.3 mL/g (in the control) to 170 mL/g when using protein-derived glycine. Meanwhile, Ponzelli et al. [27] demonstrated that GO dosage of 100–500 mg/L inhibited the AD process of microcrystalline cellulose used as a substrate in absence of antibiotics. The negative effects of GO are primarily associated with physical cell membrane damage and oxidative stress [8]. The sharp and thin edges of GO particles can physically disrupt microbial cell membranes, while the bioreduction of GO promotes the formation of reactive oxygen species, increasing oxidative stress and impairing cellular activity [1]. This is especially harmful to bacteria, as membrane damage may result in the leakage of intracellular contents (e.g., ribonucleic acid) and ultimately lead to cell death [39]. Overall, excessive dosages of graphene and GO can exert toxic and potentially irreversible effects on anaerobic microorganisms. However, earlier studies indicated that GO had a stronger inhibitory effect on microorganisms compared to graphene. The greater impact of GO on the microbial community compared to graphene may be attributed to the presence of functional groups such as hydroxyl (–OH), carbonyl (C=O), and carboxyl (–COOH), which enhance its interactions with biomacromolecules, heavy metals, and other contaminants, thereby increasing its toxicity toward sludge microorganisms [41]. In this study, the threshold dosage of GO causing negative effects on cumulative biogas yield over 40 days period was identified as 10 mg/g-TS (equivalent to 738 mg/L). In addition, an inhibitory effect of 5 mg/g-TS GO on biogas yield was observed from the beginning of sludge digestion until the end of the exponential growth phase (around day 20).

**Figure 4:**
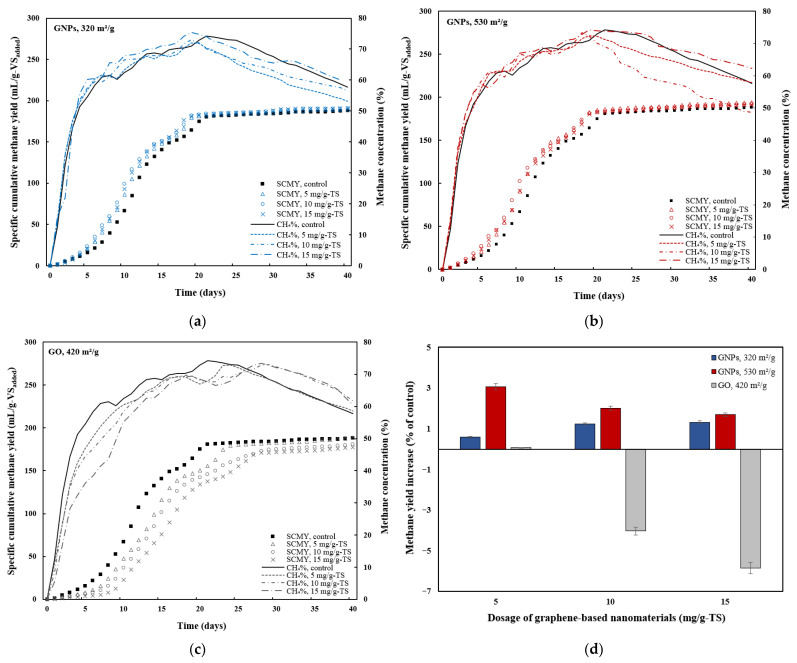
Specific cumulative methane yield and methane concentration during anaerobic digestion of thermally hydrolyzed sewage sludge amended with different dosages of (**a**) graphene nanoplatelets, 320 m^2^/g; (**b**) graphene nanoplatelets, 530 m^2^/g; (**c**) graphene oxide nanosheets, 420 m^2^/g; and (**d**) increase in methane yield for the different dosages of graphene-based nanomaterials.

The impact of various graphene-based nanomaterials (GNPs and GO) and their dosages (0, 5, 10, and 15 mg/g-TS) on specific cumulative CH_4_ yield (SCMY) and CH_4_ concentration was also investigated over a 40-day experiment (Figure 4). Methane yield increased from 188.2 mL/g-VS_added_ in the control to 194 mL/g-VS_added_ in the reactor supplemented with 5 mg/g-TS of GNPs with a surface area of 530 m^2^/g. At higher dosages of the same material (10 mg/g-TS and 15 mg/g-TS), the SCMY values were slightly lower, reaching 192 mL/g-VS_added_ and 191.4 mL/g-VS_added_, respectively (Figure 4b). In another study by Lin et al. [39], the cumulative CH_4_ yield increased from 189.3 mL/g in the control to 200.1 mL/g at a GNP dosage of 0.5 g/L when protein-derived glycine was used as the substrate. As the dosage of GNPs with a surface area of 320 m^2^/g increased from 5 mg/g-TS to 15 mg/g-TS, the CH_4_ yield gradually rose from 189.4 mL/g-VS_added_ to 190.7 mL/g-VS_added_ (Figure 4a). It can be observed that the variation in CH_4_ yield among the GNP groups with different surface areas was not significant, as the cumulative volumes differed only slightly from the control (Figure 4d). The largest difference reached only 5.78 mL/g-VS_added_ between the control and the group treated with 5 mg/g-TS of GNPs with a surface area of 530 m^2^/g. Similarly small variation in CH_4_ production was reported by Wang et al. [21], with only a 6.31 mL/g-VS_removed_ difference between the graphene treatment group and the control when a combination of SS and food waste was used as a substrate. In contrast to graphene, an increase in the amount of GO resulted in an inhibitory effect on CH_4_ yield, consistent with the trend observed in biogas production (Figure 3d). Higher dosages of GO had a significant negative effect on cumulative CH_4_ yield, which was 4.03% and 5.85% lower at 10 mg/g-TS and 15 mg/g-TS GO dosages, respectively, compared to the control. However, a low GO dosage (5 mg/g-TS) had almost no effect on CH_4_ yield, as it was only 0.59% higher compared to the control. In a similar study, Casabella-Font et al. [42] investigated the impact of monolayer GO on the AD of waste-activated sludge. The authors found that the addition of 0.025 g GO/g-VS had no significant effect on cumulative CH_4_ yield, whereas a higher dosage of 0.075 g GO/g-VS reduced cumulative specific CH_4_ production by 19 ± 3%.

In addition to SCMY, the CH_4_ content in the produced biogas was also monitored (Figure 4). The CH_4_ content in the control and GNP-treated groups increased sharply during the first six days, reaching approximately 60%. It then continued to increase gradually, reaching around 71–75% by day 19, before steadily declining to 49–62% by the end of the digestion period. Meanwhile, in the case of GO dosages ranging from 5 to 15 mg/g-TS, the increase in CH_4_ content was slower, reaching approximately 60% by days 10–12. It then gradually rose to approximately 71% by day 26 for all GO-treated groups, before declining to 59–62% by the end of the AD process. The slower increase in CH_4_ content in the GO-treated groups was likely due to the presence of oxygen-containing functional groups in GO, which may provide oxygen to methanogens and lead to increased CO_2_ generation during sludge AD [19].

It is evident that among the graphene-based nanomaterials, only GNPs exhibited a stimulatory effect on methanogenic activity, with the highest increase in SCMY of 3.08% observed in a reactor containing 5 mg/g-TS of graphene with a surface area of 530 m^2^/g (Figure 4d). Consistent with these findings, other studies have also reported that graphene positively influenced CH_4_ generation during the AD of different substrates. For example, Tian et al. [13] found that GNPs accelerated the CH_4_ production process during AD of glucose and synthetic wastewater, with dosages of 30 and 120 mg/L increasing the CH_4_ production rate by 17% and 51.4%, respectively. Meanwhile, Lin et al. [15] found that graphene enhanced the AD of ethanol, with the addition of 1 g/L graphene increasing CH_4_ yield by 25% and the production rate by 19.5%. Wang et al. [16] compared the effects of graphite, graphene, and GO on the anaerobic co-digestion of SS with food waste and found that graphene resulted in the highest increase in cumulative CH_4_ production, which was 36.09% greater than that of the control. Due to its large surface area and high electrical conductivity, graphene can enhance bacterial extracellular electron transfer and facilitate DIET, ultimately boosting CH_4_ production efficiency in AD systems. Some researchers have reported that certain microbial species involved in DIET (electrogenic microorganisms) increase in abundance in the presence of graphene-based additives. Electrogenic microorganisms are capable of benefiting from graphene-based materials by utilizing DIET mechanisms [43]. For example, Lin et al. [15] reported that electrogenic bacteria, such as *Geobacter* and *Pseudomonas*, were significantly enriched with the addition of graphene, with their relative abundances increasing to 9.9% and 6.9%, respectively. A similar increase was observed in archaeal communities, particularly *Methanobacterium* and *Methanospirillum*, whose abundances rose to 34.9% and 7.8%, respectively, in the presence of graphene. These archaea are known as hydrogenotrophic methanogens, which convert CO_2_ and H_2_ to CH_4_. Another study suggested that in situ-generated graphene could serve as a temporary electron acceptor, enabling methanogens to utilize these electrons for the reduction of CO_2_ to CH_4_ [9]. An inhibitory effect of GO on CH_4_ production was also observed by Ponzelli et al. [27], with CH_4_ yield decreasing by 18% at a GO dosage of 500 mg/L. This is also comparable to another study, which showed that the addition of 5–500 mg/L of GO reduced cumulative CH_4_ production by 2.68–17.07% during the AD of swine manure [19]. The inhibitory effect of GO on CH_4_ production was also reported in the study by Dong et al. [18], where cumulative CH_4_ production decreased by 7% and 12.6% with the addition of 54 and 108 mg/g-VS of GO to the waste-activated sludge during AD. A recent study conducted by Casabella-Font et al. [43] showed that the addition of 0.075 g/g-VS GO to a microcrystalline cellulose substrate also reduced the specific CH_4_ production by up to 15%. It was suggested that the microbial community requires a period of adaptation to the GO additive, while the additive itself may compete for electrons during its bioreduction, thereby reducing their availability for CH_4_ production. This means that the biological reduction of GO utilizes electrons from the substrate, which would otherwise contribute to CH_4_ production. Additionally, the antimicrobial activity of GO is believed to be associated with oxidative mechanisms, which are more pronounced in smaller GO sheets [44].

Toxicity of GO materials is closely related to their dosage and can arise through various mechanisms, including cell membrane perturbation, oxidative stress, or cell isolation due to wrapping [1]. Excessive GO may induce oxidative stress in microbial cells or disrupt cell membranes due to its sharp edges and surface functional groups. For example, Zhang et al. [19] speculated that a high dosage of GO exerted a wrapping effect, leading to membrane damage and oxidative stress, which in turn inhibited or killed the methanogens. Additionally, the high surface area of GO can adsorb essential nutrients, limiting their availability to methanogens. Furthermore, studies have shown that GO can inhibit the activity of coenzyme F420, which may help explain the reduction in CH_4_ production observed at higher GO dosages [18]. These combined effects may inhibit microbial activity and thus reduce CH_4_ production. Studies involving other oxide nanoparticles have also shown that the negative effect on AD is dosage-dependent. For example, Mu et al. [45] showed that a nano-ZnO dosage of 6 mg/g-TSS had no impact on CH_4_ generation during waste-activated sludge AD; however, higher dosages of nano-ZnO inhibited sludge hydrolysis, acidification, and methanation.

### 3.3. Effects of Graphene-Based Nanomaterials on Organic Matter Degradation

**Figure 5:**
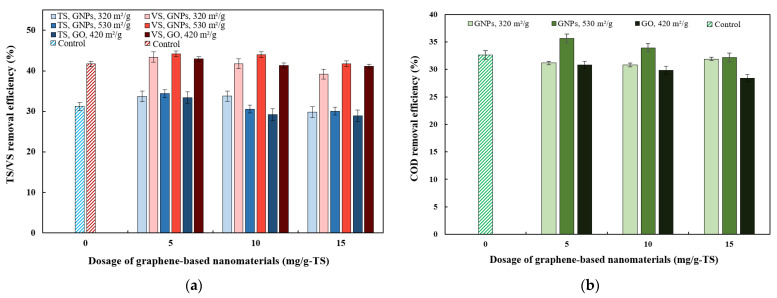
Removal efficiencies of (**a**) total solids and volatile solids, and (**b**) total chemical oxygen demand.

Changes in TS, VS, and COD were analyzed to assess the impact of graphene-based nanomaterials on the biodegradation process (Figure 5). As shown in Figure 4a and Figure 5a, TS removal and CH_4_ production followed similar patterns. The group treated with 5 mg/g-TS of GNPs with an SSA of 530 m^2^/g showed the highest TS removal (34.35%) and CH_4_ yield (194 mL/g-VS_added_), along with the greatest increase in CH_4_ production (3.08%) compared to the control. Based on other studies, more effective methods for improving TS removal efficiency exist. For example, Liu et al. [9] found that applying an electric current (1 h per day) during the AD of swine manure led to the in situ formation of graphene and increased TS removal efficiency by 9.5% compared to the group that was not exposed to electric current. Reactors B8 and B9, treated with 10 and 15 mg/g-TS of GO, respectively, showed the lowest TS removal efficiencies of 29.16% and 28.91%, both of which were lower than that of control group (31.24%). The TS removal efficiencies of groups B8 and B9 were consistent with the biogas and CH_4_ yield results, as both treatments exhibited an inhibitory effect on biogas and CH_4_ production (see Figure 3d and Figure 4d). An exception was observed with the 5 mg/g-TS dosage of GO, which resulted in a 6.04% increase in TS removal efficiency compared to the control. The inhibitory effect observed at 10 and 15 mg/g-TS GO dosages is likely due to the toxic effects of GO on microbial biomass, leading to less efficient degradation of organic matter and decreased CH_4_ generation during AD [46].

VS removal efficiencies increased by 5.52–5.97% in the groups treated with 5 and 10 mg/g-TS of GNPs (530 m^2^/g), exceeding that of the control (Figure 5a). Moreover, at equivalent dosages of 5 and 10 mg/g-TS, the GNPs with a surface area of 530 m^2^/g resulted in higher VS removal compared to those with 320 m^2^/g GNPs and GO, consistent with the corresponding trends in biogas production. The highest VS removal efficiency, 44.18%, was observed in the group with 5 mg/g-TS of GNPs with an SSA of 530 m^2^/g. This is consistent with the biogas and CH_4_ production data, which showed that the aforementioned group had the greatest impact on cumulative biogas and CH_4_ yields (see Figure 3d and Figure 4d). Meanwhile, the 5 mg/g-TS dosage of GO resulted in a 2.97% increase in VS reduction compared to the control, similar to the observed improvement in TS reduction. This GO dosage had not only a slight positive effect on VS reduction but also on cumulative biogas and CH_4_ production. Similarly, in the study by Wang et al. [21], the addition of graphene and GO slightly increased the VS removal efficiency from 75.42% in the control to 77.46% in the SS and food waste co-substrate with graphene, and to 78.82% with GO. Graphene-based materials, due to their superior properties such as high surface area and excellent electrical conductivity, create more favorable conditions for anaerobic microorganisms [47]. These conditions enhance microbial activity and growth, enabling more efficient substrate degradation. Studies have also shown that the addition of various conductive nanomaterials to anaerobic treatment systems improves the removal efficiency of TS and VS, primarily by promoting DIET, which accelerates the breakdown of organic matter [48].

The incorporation of graphene-based nanomaterials into anaerobic reactors enhances the degradation of organic matter through several pathways. First of all, it facilitates DIET, enabling microorganisms to exchange electrons intercellularly and accelerate the breakdown of organic substrates [8]. Through DIET, CH_4_ production becomes more efficient, driven by accelerated reaction kinetics and improved thermodynamic conditions [49]. DIET has shown great potential as a more stable and efficient alternative to mediated interspecies electron transfer, which relies on H_2_ and formate as intermediary “shuttle” molecules. Graphene-based materials function as catalysts and significantly contribute to improving the efficiency of substrate conversion into end products during the AD process. Secondly, it was also shown that the addition of graphene-based nanomaterials increases electron shuttling efficiency, which in turn enhances microbial proliferation [16,19].

Figure 5b shows the impact of graphene and GO on COD reduction. The highest COD removal, 35.64%, was observed in the treatment group with 5 mg/g-TS GNPs (530 m^2^/g) and it was slightly higher compared to the control (32.6%). However, the treatments did not show any notable differences in terms of COD removal efficiency. According to the literature, other conductive materials—such as biochar derived from herbal sources—also did not show a significant impact on COD removal efficiency, as the removal only increased from 68.1% to 72.5% [50].

**Figure 6:**
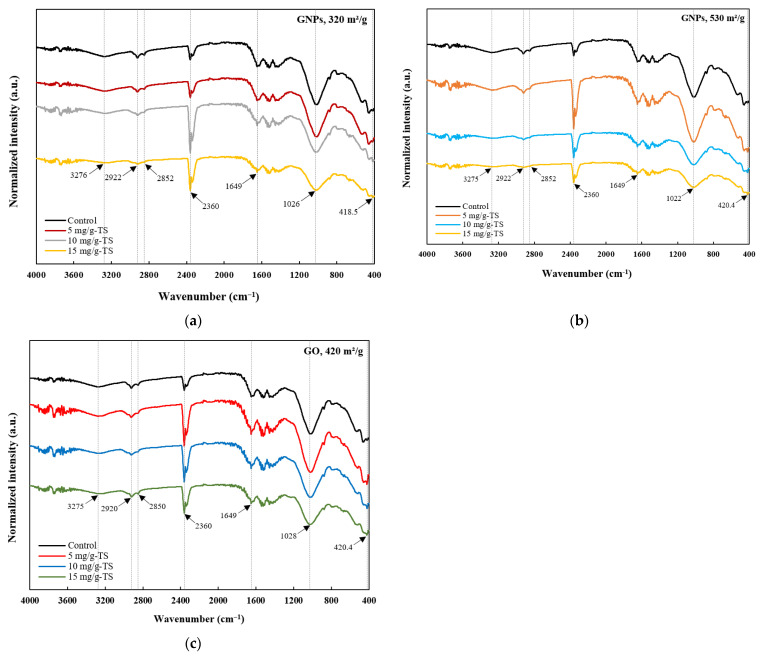
FTIR spectra of sludge digestate with (**a**) graphene nanoplatelets, 320 m^2^/g; (**b**) graphene nanoplatelets, 530 m^2^/g; (**c**) graphene oxide nanosheets, 420 m^2^/g.

The FTIR spectra of different treatment groups with and without graphene-based nanomaterials after AD are compared in Figure 6. The peaks around 3275 cm^−1^ are associated with –OH and amide (–NH) groups. That particular peak indicates the presence of –OH and –NH functional groups, which may originate from both water and organic matter present in anaerobically digested sludge [51]. It is generally accepted that the peaks at 3100–3500 cm^−1^ correspond to the vibrations of –OH and –NH groups [52,53]. NH group vibrations indicate the presence of amines and amides. The two narrow transmittance bands observed between 2850 and 2922 cm^−1^ were associated with the aliphatic symmetric and asymmetric stretching vibrations of methyl (–CH_3_) and methylene (–CH_2_) groups. It is in agreement with a previously published study, which analyzed the FTIR spectra of biogas residue [54]. The intensities of these peaks decreased with the decreasing concentration of GO, which confirms that at lower GO concentrations, organic fatty hydrocarbons were more effectively degraded into CH_4_ and other gases (Figure 6c). The band at 2360 cm^−1^ may be attributed to P–H stretching vibrations characteristic of phosphine compounds [55]. It is widely accepted that phosphine forms as a result of the breakdown of organic phosphorus compounds by certain bacteria in anaerobic environments [56]. One study also showed that during the AD of livestock manure, the amount of organic phosphorus—which included up to 41% of total phosphorus—decreased due to phosphorus uptake by microorganisms or its conversion into phosphine [57]. The peak at 1649 cm^−1^ might be associated with C=O and carboxylate (–COO) stretching vibrations of carboxylic acids, as well as aromatic annulus (C=C) stretching and –NH stretching vibrations of aromatics and olefins [58]. It can be observed that with increasing dosages of GO, the intensity of the C=O peak slightly increased, indicating the stability of this functional group and its potential toxic effect on microorganisms. It could also be speculated that the amide functional group in control sludge was more decomposed compared to the groups treated with graphene-based nanomaterials [59]. The peak around 1026 cm^−1^ was attributed to C–O and C–O–C stretching vibrations. It can be observed that the highest dosages of GO exhibited more intense C–O peaks, suggesting their potentially greater stability and toxic effect on microorganisms, which may have led to lower gas and CH_4_ yields. According to Jin et al.’s [55] study, SS is rich in oxygen-containing macromolecular aromatic compounds, which contribute to the relative stability of aromatic C–O groups. Furthermore, among all the graphene-based nanomaterials at a dosage of 15 mg/g-TS, the FTIR transmittance bands for GO (Figure 6c) were generally more well defined and intense, indicating a higher abundance of oxygen-containing functional groups in this treatment group.

### 3.4. Kinetic Modeling Results

**Figure 7:**
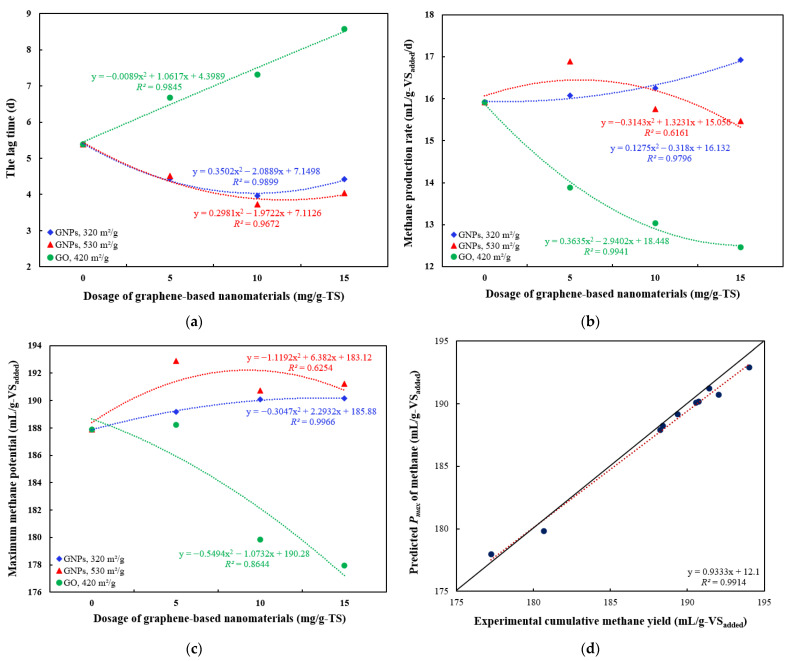
Relationships between dosages of graphene-based nanomaterials and various kinetic parameters in anaerobic digestion of thermally hydrolyzed sewage sludge: (**a**) the lag phase time, d; (**b**) maximum methane production rate, mL/g-VS_added_/d; (**c**) maximum methane potential, mL/g-VS_added_; and (**d**) scatter plot comparing measured and predicted maximum methane potential values using modified Gompertz model (linear regression model).

**Figure 8:**
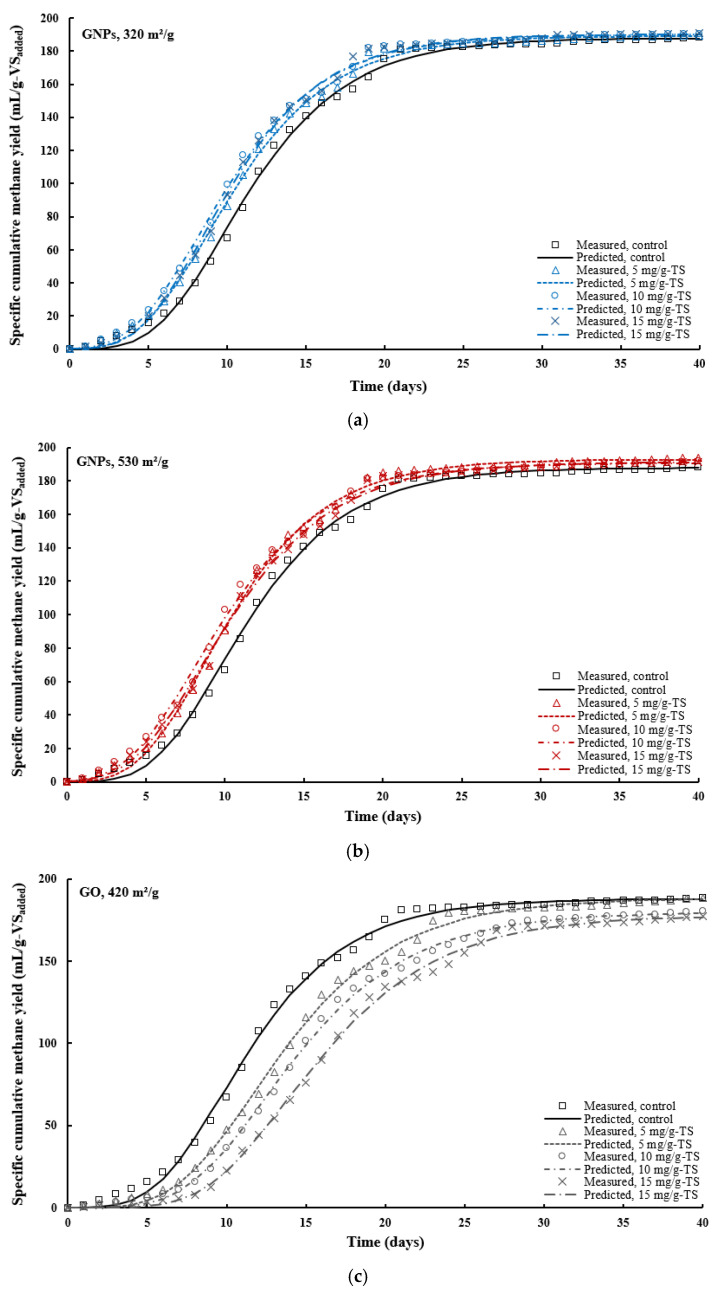
Comparison of the experimental dynamic methane yields with those predicted by the modified Gompertz model for thermally hydrolyzed sewage sludge treated with different dosages of graphene-based nanomaterials: (**a**) graphene nanoplatelets, 320 m^2^/g; (**b**) graphene nanoplatelets, 530 m^2^/g; (**c**) graphene oxide nanosheets, 420 m^2^/g.

**Figure 9:**
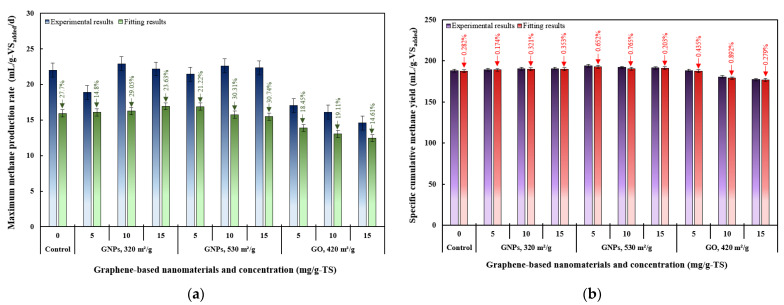
Comparison of experimental and model-predicted values from the anaerobic digestion of thermally hydrolyzed sewage sludge with different types and dosages of graphene-based nanomaterials: (**a**) maximum methane production rates, mL/g-VS_added_/d; (**b**) specific cumulative methane yields, mL/g-VS_added_.

To assess the impact of added graphene-based materials on CH_4_ production, the MGM was used to simulate the kinetics of CH_4_ generation, and the fitting results were presented in Figure 7, Figure 8 and Figure 9 and Table 3. The MGM equation (Equation (1)) was applied to the experimental data on specific cumulative CH_4_ yield to determine three kinetic parameters—maximum specific cumulative CH_4_ production as *P_max_* (mL/g-VS_added_), maximum CH_4_ production rate as *R_max_* (mL/g-VS_added_/d), and lag phase as *λ* (days)—characterizing CH_4_ generation, thereby enabling a quantitative comparison of the different conditions examined. The model was extensively applied to illustrate the dynamics of substrate consumption and bacterial growth during batch anaerobic digestion [17].

The selected model was used to simulate CH_4_ production under different dosages of graphene-based nanomaterials, yielding three kinetic parameters for comparative analysis: *P_max_*, *R_max_,* and *λ* (Figure 7). The *λ* shows the time microorganisms need to adapt to new environment and it plays a significant role in assessing AD performance [60]. The λ value of control group was 5.39 days and was reduced by up to 30.8% at a 10 mg/g-TS concentration of GNPs with an SSA of 530 m^2^/g (Figure 7a). A similar reduction in the lag phase—up to 26.5%—was also observed at the same concentration of GNPs with a smaller surface area (320 m^2^/g). Statistically significant differences in λ values were observed between both treatment groups and the control (*p* < 0.05). This is consistent with the results of another study, where a graphene dosage of 0.5 g/L resulted in a lag phase of 3.45 days [15]. A short lag phase is commonly linked to effective CH_4_ generation [22]. The low λ values suggest that CH_4_ generation started quickly, likely due to the rapid hydrolysis of organic material, which increased the availability of VFAs for methanogens, resulting in more efficient CH_4_ production [60]. Thus, the proper dosage of GNPs increased the CH_4_ production peak and improved the overall performance of the AD process. On the other hand, it was observed that increasing the dosage of GO led to a longer lag phase, and this relationship was linear. The lag phase values in the 5, 10, and 15 mg/g-TS GO groups were 1.24, 1.36, and 1.59 times higher compared to the control. This was consistent with the findings of Casabella-Font [43], which showed that the addition of GO resulted in a lag phase that was twice as long as that of the control. Increased λ values imply that GO extended the lag phase and hindered the hydrolysis process, likely due to biological reduction.

Figure 7d presents a visual comparison between the experimentally observed cumulative CH_4_ yields and the model-predicted values, depicting the correlation between the obtained data and the model’s predictions. The dashed line indicates the linear fit. The linear regression of specific cumulative CH_4_ yield values is expected to align with the 1:1 line of perfect fit (solid line), indicating no bias. As seen in the plot, the points are closely distributed along a straight dashed line, indicating that the MGM fits the data well. The close proximity of most points to the 45° line reflects the model’s high accuracy [61]. This also indicates that the residuals follow a normal distribution. The MGM demonstrated over 90% agreement with the observed data, as indicated by a coefficient of determination greater than 0.9 (*R*^2^ > 0.99).

To assess how well the MGM fits the kinetic data, the coefficient of determination (*R*^2^), along with the calculated *RMSE* and *NRMSE* values between the experimental and predicted results, were evaluated (Table 3). According to the statistical metrics, all treatment groups demonstrated satisfactory accuracy in predicting cumulative methane production. The highest *R*^2^ values (>0.998) were observed in the GO treatment groups. The *R*^2^ values obtained for all treatment groups were close to each other, ranging from 0.986 to 0.999, indicating that the selected model has a strong ability to explain the majority of the data variation. Additionally, an *R*^2^ value approaching 1 indicates a higher level of accuracy in the model’s simulation performance. The *RMSE* and *NRMSE* values indicate the deviation between the actual CH_4_ values and those predicted by the model for the different treatment groups. For all treatment groups, *RMSE* values ranged from 2.441 mL/g-VS_added_ to 3.302 mL/g-VS_added_, and *NRMSE* values from 1.351% to 1.754%. It can be seen that the *NRMSE*, representing the fitting error, was below 2% for all groups, indicating excellent agreement between predicted and observed CH_4_ yields. The most accurate CH_4_ production prediction was obtained in reactor B8, treated with 10 mg/g-TS GO, due to the highest *R*^2^ (0.999) value and the lowest *RMSE* and *NRMSE* values (2.441 mL/g-VS_added_ and 1.351%). The MGM proved to be a reliable tool for predicting CH_4_ production during the AD of THSS with graphene-based nanomaterials. The findings of the present study are consistent with a previous study, where experimental specific CH_4_ production data closely matched the values predicted by the MGM when *R*^2^ of 1 and relative *RMSE* values of 1.5% were obtained for microcrystalline cellulose [62]. In another study, Almegbl et al. [63] also observed a strong agreement between the experimental data of cumulative methane yield and the *P_max_* predicted by the MGM for AD of thermo-chemically pretreated sludge supplemented with graphene material. The *R*^2^ values exceeded 0.99, indicating excellent fit between the experimental data and model.

Figure 8 shows a comparison between the experimentally measured cumulative methane yields and those estimated using the MGM over the 40-day AD of THSS supplemented with different concentrations of graphene-based nanomaterials. It can be seen that the selected model is suitable for predicting dynamics of CH_4_ production. The CH_4_ generation trend in the fitting curves can be divided into three distinct phases: (1) a lag phase, (2) an exponential growth phase, and (3) a stationary phase. Initially, a lag phase lasting approximately 4–7 days was observed, reflecting the time required for microbial adaptation and the establishment of AD conditions. This was followed by a phase of rapid CH_4_ generation, which lasted from around day 4–7 to approximately day 20. Finally, a stabilization phase occurred from day 20 until the end of the AD process. In another study conducted by Adelard et al. [64], a similar pattern was observed, where the co-digestion of pig manure, cow manure, and food waste resulted in an initial digestion phase from day 0 to 7, an intermediate phase from day 7 to 20, and a final phase from day 20 until the end of the AD process on day 36.

In all groups, the experimental *R_max_* values were higher than those obtained from the model fitting (Figure 9a). Among the groups, the treatment with 15 mg/g-TS GNPs of 320 m^2^/g surface area yielded the highest fitted *R_max_* value (16.92 mL/g-VS_added_/d), while the 15 mg/g-TS GO group showed the lowest (12.46 mL/g-VS_added_/d). Meanwhile, the highest experimental *R_max_* value (22.91 mL/g-VS_added_/d) was recorded in the group treated with 10 mg/g-TS of GNPs with a surface area of 320 m^2^/g, and the lowest was observed in the group with 15 mg/g-TS of GO (14.59 mL/g-VS_added_/d). These results indicate that model fitting showed partial agreement with the experimental methane production rates.

Figure 9b presents the specific cumulative CH_4_ yields from AD of THSS amended with graphene-based nanomaterials, as determined by both the MGM and experimental data. For all treatment groups, the SCMY values predicted by the model were slightly lower than the experimentally observed values. The difference between the experimental and simulated values was within 1%, demonstrating strong agreement between the predicted and actual results. The highest experimental and fitted *P_max_* values (194 and 192.9 mL/g-VS_added_, respectively) were observed in the group treated with 5 mg/g-TS of GNPs (530 m^2^/g), while the lowest (177.2 and 177.9 mL/g-VS_added_, respectively) were observed in the group treated with 15 mg/g-TS of GO. These results confirm that the cumulative CH_4_ yield curves for all treatment groups followed a sigmoidal pattern, consistent with the three-phase model described by the MGM.

## 4. Conclusions

This study examined the effects of GNPs and GO nanosheets, differing in SSA, on biogas and methane production as well as kinetic behavior during the anaerobic digestion of thermally hydrolyzed sewage sludge. Graphene nanoplatelets of both SSAs enhanced biogas production at 5 and 10 mg/g-TS dosages, with those having the larger SSA (530 m^2^/g) showing greater effectiveness, resulting in increases of 3.87% and 3.76% compared to the control, respectively. Regarding methane production, GNPs with a larger SSA also demonstrated higher efficiency, resulting in a 3.08% increase at a 5 mg/g-TS dosage compared to the control. Additionally, kinetic modeling results showed that GNPs with an SSA of 530 m^2^/g at 5 mg/g-TS dosage led to the highest methane potential of 192.9 mL/g-VS_added_, one of the highest methane production rates (16.89 mL/g-VS_added_/d) and one of the greatest reductions in the lag phase duration compared to the control (from 5.39 to 4.51 days), respectively. Therefore, 5 mg/g-TS of GNPs with a larger SSA played a key role in boosting biogas and methane production from thermally hydrolyzed sewage sludge, yielding a maximum of 316.7 mL biogas/g-VS_added_ and 194 mL CH_4_/g-VS_added_. The analysis of organic matter degradation efficiency was consistent with the biogas and methane production results, as the highest total and volatile solids removals (34.35% and 44.18%, respectively) were observed in the experimental group treated with 5 mg/g-TS GNPs having an SSA of 530 m^2^/g. This suggests that the GNPs served as electron shuttles and facilitated DIET. Improvements in CH_4_ production during the AD of THSS supplemented with GNPs observed in lab-scale experiments suggest that incorporating GNPs could be a promising strategy to enhance the efficiency of industrial-scale steady-state AD systems. The accelerated CH_4_ production and shortened lag phase associated with low-dose GNP application may lead to increased treatment efficiency within shorter retention times, potentially enabling a reduction in reactor volume requirements and operational costs. Therefore, the use of higher-surface-area GNPs at a low dose (5 mg/g-TS) is recommended for the AD of THSS in wastewater treatment plants.

## Figures and Tables

**Table 1:** Main characteristics of substrate, inoculum, and their mixture (mean ± standard deviation; *n* = 3).

Parameter	Substrate	Inoculum	Mixture
Total solids (TS, %)	8.85 ± 0.14	4.59 ± 0.03	7.38 ± 0.05
Volatile solids (VS, %)	4.08 ± 0.03	2.64 ± 0.01	3.62 ± 0.01
VS/TS (%)	46.10	57.52	49.05
Total chemical oxygen demand (COD, mg/L)	70 912 ± 1281	45 133 ± 405	62 319 ± 597
Electrical conductivity (mS/cm)	2.45 ± 0.02	10.13 ± 0.02	5.36 ± 0.03
pH	5.26 ± 0.09	7.615 ± 0.05	7.009 ± 0.004
C (%)	25.33 ± 0.12	23.8 ± 0.10	24.82 ± 0.11
N (%)	3.72 ± 0.07	3.61 ± 0.06	3.69 ± 0.07
C/N	6.81	6.59	6.73

**Table 2:** Experimental design.

Reactor Code	Added Substrate Amount (kg)	Added Inoculum Amount (kg)	Type of Graphene-Based Nanomaterial	Specific Surface Area, m^2^/g	Added Amount of Graphene-Based Nanomaterial (g)	Dosage of Graphene-Based Nanomaterial (mg/g-TS)
B0	1.334	0.667	-	-	0	0
B1			Graphene nanoplatelets (GNPs)	320	0.738	5
B2					1.476	10
B3					2.214	15
B4			Graphene nanoplatelets (GNPs)	530	0.738	5
B5					1.476	10
B6					2.214	15
B7			Graphene oxide (GO) nanosheets	420	0.738	5
B8					1.476	10
B9					2.214	15

**Table 3:** Statistical parameters for modified Gompertz model evaluation.

Statistical Parameter	Reactor Code									
	B0	B1	B2	B3	B4	B5	B6	B7	B8	B9
*R* ^2^	0.994	0.989	0.986	0.988	0.989	0.986	0.987	0.998	0.999	0.999
*RMSE*, mL/g-VS_added_	3.302	2.829	2.839	2.608	2.915	3.132	3.021	3.092	2.441	2.656
*NRMSE*, %	1.754	1.494	1.489	1.367	1.502	1.631	1.578	1.641	1.351	1.499

## Data Availability

The data supporting the findings of this study can be obtained from the corresponding author upon reasonable request.

## Abbreviations

The following abbreviations are used in this manuscript:

GNPs	Graphene nanoplatelets
GO	Graphene oxide
SS	Sewage sludge
THSS	Thermally hydrolyzed sewage sludge
AD	Anaerobic digestion
WWTP	Wastewater treatment plant
SEM	Scanning electron microscopy
EDS	Energy-dispersive spectroscopy
VFAs	Volatile fatty acids
DIET	Direct interspecies electron transfer
GAC	Granular activated carbon
CNPs	Carbonaceous nanoparticles
TS	Total solids
VS	Volatile solids
COD	Chemical oxygen demand
SSA	Specific surface area
MGM	Modified Gompertz model
*RMSE*	Root mean square error
*NRMSE*	Normalized root mean square error
SCBY	Specific cumulative biogas yield
SCMY	Specific cumulative methane yield
FTIR	Fourier transform infrared
